# Latitude and HLA-DRB1*04:05 independently influence disease severity in Japanese multiple sclerosis: a cross-sectional study

**DOI:** 10.1186/s12974-016-0695-3

**Published:** 2016-09-06

**Authors:** Yuri Nakamura, Takuya Matsushita, Shinya Sato, Masaaki Niino, Toshiyuki Fukazawa, Satoshi Yoshimura, Shin Hisahara, Noriko Isobe, Shun Shimohama, Mitsuru Watanabe, Kazuto Yoshida, Hideki Houzen, Yusei Miyazaki, Ryo Yamasaki, Seiji Kikuchi, Jun-ichi Kira, the Japan Multiple Sclerosis Genetics Consortium

**Affiliations:** 1 Department of Neurology, Neurological Institute, Graduate School of Medical Sciences, Kyushu University, 3-1-1 Maidashi, Higashi-ku, Fukuoka 812-8582 Japan; 2 Department of Clinical Research, Hokkaido Medical Center, Yamanote 5-jo 7-chome, Nishi-ku, Sapporo 063-0005 Japan; 3 Sapporo Neurology Clinic, 21-2-1, Kita 21-jo Higashi, Higashi-ku, Sapporo 065-0021 Japan; 4 Department of Neurology, School of Medicine, Sapporo Medical University, South 1 West 16, Chuo-ku, Sapporo 060-8543 Japan; 5 Department of Neurology, Asahikawa Red Cross Hospital, 1-1-1, Akebono 1-jo, Asahikawa, 070-8530 Japan; 6 Department of Neurology, Obihiro Kosei General Hospital, 8-1, Nishi 6-jo Minami, Obihiro, 080-0016 Japan; 7 Department of Neurology, Hokkaido Medical Center, Yamanote 5-jo 7-chome, Nishi-ku, Sapporo 063-0005 Japan

**Keywords:** Multiple sclerosis, *HLA*, Latitude, Magnetic resonance imaging, Oligoclonal IgG bands

## Abstract

**Background:**

Higher latitude and human leukocyte antigen (*HLA*)-DRB1*04:05 increase susceptibility to multiple sclerosis (MS) in the Japanese population, but their effects on disease severity are unknown. We aimed to clarify the effects of latitude and the *HLA-DRB1* and *HLA-DPB1* genes on disease severity in Japanese patients with MS.

**Methods:**

We enrolled 247 MS patients and 159 healthy controls (HCs) from the northernmost main island of Japan, Hokkaido Island (42–45° north), and 187 MS patients and 235 HCs from the southern half (33–35° north) of the Japanese archipelago (33–45° north). We genotyped *HLA-DRB1* and *HLA-DPB1* alleles, compared demographic features, and analyzed factors contributing to differences in clinical and laboratory findings between MS patients from southern and northern Japan. The Multiple Sclerosis Severity Score (MSSS), which adjusts the Kurtzke’s Expanded Disability Status Scale score according to disease duration, was used to estimate disease severity.

**Results:**

The HLA-DRB1*04:05 and DRB1*15:01 alleles conferred susceptibility to MS in our Japanese population (*p*^corr^ = 0.0004 and *p*^corr^ = 0.0019, respectively). Southern patients had higher MSSS scores than northern patients (*p* = 0.003). Northern patients had higher frequencies of brain lesions meeting the Barkhof criteria (Barkhof brain lesions) and cerebrospinal fluid (CSF) IgG abnormalities than southern patients (*p* = 0.0012 and *p* < 0.0001, respectively). DRB1*04:05-positive MS patients had lower MSSS scores and lower frequencies of Barkhof brain lesions and CSF IgG abnormalities than DRB1*04:05-negative MS patients (*p* = 0.0415, *p* = 0.0026, and *p* < 0.0001, respectively). Multivariate analyses revealed that latitude and DRB1*04:05 were independently associated with the lowest quartile of MSSS and that latitude was positively associated with Barkhof brain lesions and CSF IgG abnormalities. DRB1*04:05 was negatively associated with these parameters. MSSS was decreased by 0.57 per DRB1*04:05 allele (*p* = 0.0198).

**Conclusions:**

Living at a higher latitude and carrying the DRB1*04:05 allele independently lessens MS symptom severity as defined by MSSS. However, these factors influence the frequency of Barkhof brain lesions and CSF IgG abnormalities in opposite ways; higher latitude increases the frequency of Barkhof brain lesions and CSF IgG abnormalities, whereas DRB1*04:05 decreases them.

**Electronic supplementary material:**

The online version of this article (doi:10.1186/s12974-016-0695-3) contains supplementary material, which is available to authorized users.

## Background

Multiple sclerosis (MS) is a chronic disease of the central nervous system characterized by inflammation, demyelination, and axonal injury [1]. MS susceptibility and phenotypes result from complex interactions between multiple genetic and environmental factors [2]. It is well established that the class II subregion of the human leukocyte antigen (*HLA*) gene on chromosome 6p21 plays the most important role in determining MS susceptibility [3]. The *DR15* haplotype (DRB1*15:01-DQA1*01:02-DQB1*06:02) is strongly associated with MS, and the effect is mainly driven by HLA-DRB1*15:01 in people of northern European descent [4, 5]. Although *DRB1* alleles clearly influence MS susceptibility, it is not known how *HLA* variation influences the clinical features of MS, especially in Asian populations.

We previously reported that the HLA-DRB1*04:05 allele is the strongest genetic risk factor for MS in the southern Japanese population. The clinical phenotypes of patients with the HLA-DRB1*04:05 allele include a milder clinical course, younger age at disease onset, slower disease progression, fewer brain lesions, and rare cerebrospinal fluid (CSF) IgG abnormalities compared with patients lacking the HLA-DRB1*04:05 allele [6]. It is widely known that the risk for MS increases with distance from the equator [7], suggesting that environmental risk factors related to latitude, such as decreased sunlight exposure and vitamin D levels, are involved [8]. Additionally, despite genetic homogeneity, there are phenotypic differences between northern and southern Japanese patients with MS. In the fourth nationwide survey in Japan, brain magnetic resonance imaging (MRI) findings fulfilling the Barkhof criteria [9] were significantly more common in northern patients than in southern patients and more frequent in northern-born northern residents than northern-born southern residents [10]. Recent studies in Western countries have revealed an association between latitude and the presence of oligoclonal IgG bands (OB) or increased intrathecal production of IgG [11, 12]. We have also reported that latitude independently affects the emergence of CSF IgG abnormalities in Japanese patients with MS [13].

These observations suggest that latitude may influence the clinical phenotype of MS. However, the relationship between latitude and disease severity still remains unclear. Thus, the aim of this study was to clarify the influence of latitude and *HLA*-*DRB1* on disease severity in Japanese patients with MS.

## Methods

### Participants

Patients with MS, agreeing to participate in this study, were recruited from nine institutes in Japan. MS was diagnosed using the 2010 revised McDonald criteria [14]. Patients with neuromyelitis optica [15], neuromyelitis optica spectrum disorders [16], or longitudinally extensive spinal cord lesions extending over three or more vertebral segments were excluded. Patients with primary progressive MS (PPMS) were also excluded because of the low prevalence of PPMS in the Japanese population [10], as well as the association between PPMS and distinct *HLA* class II alleles from relapse-onset MS [17, 18]. Participants were recruited from the northern and southern parts of the Japanese archipelago, which spans 33–45° north (Additional file 1: Figure S1). Patients recruited from Hokkaido Island, the northernmost main island of Japan (42–45° north), were classified as the northern group, and those recruited from the southern half of the Japanese archipelago (33–35° north) were classed as the southern group. Patients residing on Hokkaido were chosen for the northern group because most people living on Hokkaido are descendants of immigrants who came from the other main islands 100–150 years ago, during the Meiji era, and thus share similar genetic makeup with modern inhabitants of the other main islands [10]. These populations were considered suitable for studying the effects of latitude, because of the latitude difference of at least 7° between Hokkaido Island and southern Japan. In total, 247 patients with MS and 159 healthy controls (HCs) in the northern group, and 187 patients with MS and 235 HCs in the southern group, participated in this study. Although sex and age were not matched between MS patients and HCs, HCs were used only for comparing *HLA* phenotypic frequency. Therefore, we believe that the use of unmatched HCs would not distort our results. We collected the demographic and clinical data of the participants including sex, age, age at disease onset, disease duration, MS subtype, annualized relapse rate (ARR), Kurtzke’s Expanded Disability Status Scale (EDSS) [19] score, and Multiple Sclerosis Severity Score (MSSS) [20] at the time of DNA blood sampling. Fulfillment of the Barkhof criteria for MS [9], positivity of CSF OB (determined by isoelectric focusing) [21], and abnormality of IgG index as calculated by (CSF IgG/serum IgG)/(CSF albumin/serum albumin) were determined by retrospective review of each participant’s medical records. In all patients, the presence of OB in CSF was assessed in a single laboratory (LSI Medience Corporation, Tokyo, Japan) and EDSS scores measured during remission were used. MSSS, which adjusts the EDSS score according to disease duration, was used to estimate the disease severity for each individual patient on a cross-sectional basis [20]. We defined the presence of Barkhof brain lesions by fulfillment of the four dichotomized MRI parameters proposed by Barkhof et al.: (1) the presence of at least one gadolinium-enhancing lesion or nine T2 hyperintense lesions, (2) the presence of at least one infratentorial lesion, (3) the presence of at least one juxtacortical lesion, and (4) the presence of at least three periventricular lesions [9]. We included cerebellar lesions as infratentorial lesions in the criteria. The IgG index was considered to be elevated if it was >0.658 [22]. We defined secondary progressive MS (SPMS) as a sustained increase in ≥1 point when EDSS was ≤5.5 and as a sustained increase in EDSS of ≥0.5 point when EDSS was ≥6.0 [23]. Benign MS was defined by an EDSS score of ≤2.0 at least 10 years after disease onset [24]. Patients and HCs were recruited from June 2012 to January 2013 in the northern group and from April 2006 to January 2013 in the southern group [6]. As the prevalence of MS is lower in southern Japan than in northern Japan [25], a longer recruitment period was allowed in the southern group. The sample sizes required to reveal an association of the HLA-DRB1*04:05 allele with MS were calculated to be 122 cases and 122 controls, based on our previous report [6]. We also collected clinical data from 109 of the 187 southern patients up to January 2013 to adjust for the different recruitment periods between the groups. The ethics committees of each institution approved this study, and written informed consent was obtained from all participants.

### *HLA-DRB1* and *HLA-DPB1* genotyping

The genotypes of the participants *HLA-DRB1* and *HLA-DPB1* alleles were determined by hybridization between polymerase chain reaction amplification products of the *HLA-DRB1* and *HLA-DPB1* genes and sequence-specific oligonucleotide probes, as previously described [26].

### Statistical analysis

The phenotypic frequencies of the *HLA-DRB1* and *HLA-DPB1* alleles were compared using either the chi-square test or Fisher’s exact probability test (when criteria for the chi-square test were not fulfilled). The chi-square test was used to compare sex, SPMS, brain MRI lesions meeting the Barkhof criteria, and CSF IgG abnormalities between subgroups. We performed the Shapiro–Wilk test for normality for continuous variables, such as age, age at onset, disease duration, ARR, EDSS, and MSSS. As these continuous variables were not normally distributed, we used the Mann–Whitney *U* test for comparisons between subgroups. The Mann–Whitney *U* test was also used to compare MSSS between patients with and without Barkhof brain lesions and between patients with and without CSF IgG abnormalities.

A multivariate linear regression model was used for continuous dependent variables (log-transformed age at onset), while a logistic regression model was used for binary dependent variables (benign MS, fulfillment of Barkhof criteria for MS, and CSF IgG abnormalities), adjusting for other factors associated with dependent variables. We divided MSSS into quartiles defined by MSSS values of 0.94, 2.60, and 5.50, and a logistic regression model was used. All analyses were performed using JMP 11.0.0 (SAS Institute Inc., Cary, NC, USA). Statistical significance was set at *p* < 0.05. Uncorrected *p* values (*p*^uncorr^) were corrected by multiplying them by the number of comparisons (Bonferroni–Dunn’s correction) to calculate corrected *p* values (*p*^corr^).

## Results

### Demographic features

The clinical features of the 434 patients with MS (314 females, 120 males) are summarized in Table 1. The median age at disease onset was 29 years (interquartile range 22–38 years). The frequency of SPMS was 17.1 %. The median EDSS score was 2.0 (interquartile range 1.0–3.5), and the median MSSS was 2.60 (interquartile range 0.94–5.50). A total of 318 patients (74.3 %) had Barkhof brain lesions, and 193 patients (58.7 %) had CSF IgG abnormalities.

**Table 1:** Demographic features of patients with MS according to region

	Total patients (*n* = 434)	Northern patients (*n* = 247)	Southern patients (*n* = 187)	*p* value
Number of males/females (ratio)	120/314 (1:2.6)	62/185 (1:3.0)	58/129 (1:2.2)	NS
Age (years)^a^	41 (33–50)	41 (34–50)	40 (32–50)	NS
Age at onset (years)^a^	29 (22–38)	29 (22–36)	30 (23–40)	NS
SPMS (%)	73/427 (17.1)	53/245 (21.6)	20/182 (11.0)	0.0039
Disease duration (years)^a^	9 (4–15)	10 (6–17)	6.5 (3–13)	<0.0001
EDSS^a^	2.0 (1.0–3.5)	2.0 (1.0–3.5)	2.0 (1.0–3.5)	NS
MSSS^a^	2.60 (0.94–5.50)	2.13 (0.69–5.24)	3.54 (1.45–5.90)	0.003
ARR^a^	0.41 (0.22–0.75)	0.41 (0.24–0.73)	0.40 (0.20–0.89)	NS
Barkhof criteria (%)	318/428 (74.3)	198/247 (80.2)	120/181 (66.3)	0.0012
Positive OB and/or increased IgG index (%)	193/329 (58.7)	130/177 (73.5)	63/152 (41.5)	<0.0001
Phenotypic frequency of HLA-DRB1*04:05 (%)	172 (39.6)	92 (37.3)	80 (42.8)	NS
Phenotypic frequency of HLA-DRB1*15:01 (%)	125 (28.8)	75 (30.4)	50 (26.7)	NS

The Mann–Whitney *U* test was used to compare continuous variables, and the chi-square test was used to compare categorical variables

*ARR* annualized relapse rate, *EDSS* Kurtzke’s Expanded Disability Status Scale, *MSSS* Multiple Sclerosis Severity Score, *NS* not significant, *OB* oligoclonal IgG bands, *SPMS* secondary progressive multiple sclerosis

^a^Median (interquartile range)

### Frequencies of the *HLA-DRB1* and *HLA-DPB1* alleles

As shown in Table 2, the phenotypic frequencies of the DRB1*04:05 and DRB1*15:01 alleles were significantly higher in MS patients than HCs (*p*^corr^ = 0.0004 and *p*^corr^ = 0.0019, respectively). The phenotypic frequencies of DRB1*01:01, DRB1*09:01, DRB1*13:02, and DRB1*15:02 were significantly lower in MS patients than HCs (*p*^corr^ = 0.003, *p*^corr^ = 0.0198, *p*^corr^ = 0.0066, and *p*^corr^ = 0.0396, respectively). No *DPB1* alleles were associated with MS in Japan (Additional file 2: Table S1). Compared with HCs, the phenotype frequency of DRB1^*^04:05 was significantly higher in patients with MS in both northern and southern regions (*p*^corr^ = 0.0224 and *p*^corr^ = 0.0384, respectively) (Additional file 2: Tables S2 and S3). There was no significant difference in the distribution of *DRB1* alleles between northern and southern HCs (Additional file 2: Table S4).

**Table 2:** Phenotypic frequencies of *HLA-DRB1* alleles in patients with MS

*DRB1*	Phenotype frequency, *n* (%)		OR	95 % CI	*p* ^uncorr^	*p* ^corr^
	MS (*n* = 434)	HCs (*n* = 394)				
01:01	24 (5.5)	53 (13.5)	0.38	0.23–0.62	<0.0001	0.003
03:03	1 (0.2)	0	NA	NA	1.0000	NS
04:01	7 (1.6)	9 (2.3)	0.70	0.26–1.90	0.6151	NS
04:03	42 (9.7)	22 (5.6)	1.81	1.06–3.09	0.0276	NS
04:04	2 (0.5)	2 (0.5)	0.91	0.13–6.47	1.0000	NS
04:05	172 (39.6)	100 (25.4)	1.93	1.43–2.60	<0.0001	0.0004
04:06	42 (9.7)	26 (6.6)	1.52	0.91–2.52	0.1071	NS
04:07	2 (0.5)	4 (1.0)	0.45	0.08–2.48	0.4317	NS
04:10	24 (5.5)	8 (2.0)	2.82	1.25–6.36	0.0091	NS
07:01	1 (0.2)	2 (0.5)	0.45	0.04–5.01	0.6073	NS
07:10	1 (0.2)	0	NA	NA	1.0000	NS
08:01	1 (0.2)	1 (0.3)	0.91	0.06–14.56	1.0000	NS
08:02	38 (8.8)	29 (7.4)	1.21	0.73–2.00	0.4622	NS
08:03	63 (14.5)	56 (14.2)	1.02	0.69–1.51	0.9012	NS
09:01	72 (16.6)	104 (26.4)	0.55	0.40–0.78	0.0006	0.0198
10:01	2 (0.5)	2 (0.5)	0.91	0.13–6.47	1.0000	NS
11:01	18 (4.2)	19 (4.8)	0.85	0.44–1.65	0.6388	NS
12:01	26 (6.0)	38 (9.6)	0.60	0.36–1.00	0.0493	NS
12:02	5 (1.2)	15 (3.8)	0.29	0.11–0.82	0.0211	NS
13:01	3 (0.7)	2 (0.5)	1.36	0.23–8.21	1.0000	NS
13:02	20 (4.6)	46 (11.7)	0.37	0.21–0.63	0.0002	0.0066
13:07	0	1 (0.3)	0	NA	0.4758	NS
14:01	3 (0.7)	0	NA	NA	0.2508	NS
14:02	1 (0.2)	1 (0.3)	0.91	0.06–14.56	1.0000	NS
14:03	20 (4.6)	14 (3.6)	1.31	0.65–2.63	0.4449	NS
14:05	17 (3.9)	15 (3.8)	1.03	0.51–2.09	0.9347	NS
14:06	11 (2.5)	6 (1.5)	1.68	1.62–4.59	0.3371	NS
14:07	0	2 (0.5)	0	NA	0.2261	NS
14:54	20 (4.6)	28 (7.1)	0.63	0.35–1.14	0.1245	NS
15:01	125 (28.8)	67 (17.0)	1.97	1.41–2.76	<0.0001	0.0019
15:02	55 (12.7)	83 (21.1)	0.54	0.37–0.79	0.0012	0.0396
15:10	1 (0.2)	0	NA	NA	1.0000	NS
16:02	4 (0.9)	4 (1.0)	0.91	0.23–3.65	1.0000	NS

*p* ^*uncorr*^ was corrected by multiplying the value by 33 to calculate *p* ^corr^

*CI* confidence interval, *HCs* healthy controls, *MS* multiple sclerosis, *NA* not applicable, *NS* not significant, *OR* odds ratio, *p* ^*corr*^ corrected *p* value

### Comparison of clinical characteristics between northern and southern patients

Northern MS patients had significantly lower MSSS scores compared with southern patients (*p* = 0.003) (Table 1), although the frequency of SPMS was higher in the north than in the south (*p* = 0.0039). Northern patients had a significantly longer disease duration, a higher frequency of Barkhof brain lesions, and a higher frequency of CSF IgG abnormalities compared with southern patients (*p* < 0.0001, *p* = 0.0012, and *p* < 0.0001, respectively). Even after excluding all SPMS patients, northern patients had a significantly longer disease duration, lower EDSS and MSSS, and higher frequencies of Barkhof brain lesions and CSF IgG abnormalities compared with southern patients (*p* = 0.0004, *p* = 0.0008, *p* < 0.0001, *p* = 0.0217, and *p* < 0.0001, respectively) (Additional file 2: Table S5). Similar results were seen when comparing MS patients between northern and southern Japan. There were no significant differences in the phenotypic frequencies of *HLA-DRB1 04:05* and *HLA-DRB1 15:01* between northern and southern patients (37.3 vs. 42.8 %, *p* = 0.2432; 30.4 vs. 26.7 %, *p* = 0.4087, respectively) (Table 1). After excluding all SPMS patients, there were still no differences in the frequencies of either allele between northern and southern patients (*HLA-DRB1 04:05*, 37.6 vs. 43.7 %, *p* = 0.2403; *HLA-DRB1 15:01*, 30.9 vs. 27.5 %, *p* = 0.4816) (Additional file 2: Table S5).

When we compared the demographic features of northern and southern patients with using clinical data from southern patients that were followed up until January 2013, there were no significant differences in disease duration, EDSS, or MSSS, although SPMS frequency was significantly higher in northern Japan (*p* = 0.0256) (Additional file 2: Table S6). When we excluded SPMS patients from the MS patient group, to adjust for the different recruitment periods, the northern patients had significantly lower MSSS (median 1.45 vs. 2.23, *p* = 0.0411) (Additional file 2: Table S7).

We examined the association of MSSS with Barkhof brain lesions and CSF IgG abnormalities and found that neither was associated with MSSS in the total population nor in the northern or southern MS patient populations (Additional file 2: Table S8).

### Comparison of clinical characteristics between HLA-DRB1*04:05-positive and HLA-DRB1*04:05-negative patients

MS patients with DRB1*04:05 were younger at disease onset, had a lower MSSS score, and had lower frequencies of Barkhof brain lesions and CSF IgG abnormalities than those without DRB1*04:05 (*p* = 0.0003, *p* = 0.0415, *p* = 0.0026, and *p* < 0.0001, respectively) (Table 3). In southern MS patients, DRB1*04:05 carriers were younger and had an earlier onset, longer disease duration, lower EDSS and MSSS scores, and lower frequencies of Barkhof brain lesions and CSF IgG abnormalities than non-DRB1*04:05 carriers (*p* = 0.0334, *p* = 0.0001, *p* = 0.0133, *p* = 0.0263, *p* = 0.0021, *p* = 0.0143, and *p* = 0.008, respectively). In northern patients, DRB1*04:05 carriers also had a lower frequency of CSF IgG abnormalities than non-DRB1*04:05 carriers (*p* = 0.0007), while age at onset, MSSS, and Barkhof brain lesion frequency in northern patients showed similar trends to the southern patients, but were not significantly different between DRB1*04:05 carriers and non-DRB1*04:05 carriers.

**Table 3:** Comparison of the clinical features of patients with MS according to the presence or absence of HLA-DRB1*04:05

DRB1	Total patients			Northern patients			Southern patients		
	*04:05* (+) (*n* = 172)	*04:05* (−) (*n* = 262)	*p* value	*04:05* (+) (*n* = 92)	*04:05* (−) (*n* = 155)	*p* value	*04:05* (+) (*n* = 80)	*04:05* (−) (*n* = 107)	*p* value
Number of males/females (ratio)	52/120 (1:2.3)	68/194 (1:2.9)	NS	27/65 (1:2.4)	35/120 (1:3.4)	NS	25/55 (1:2.2)	33/74 (1:2.2)	NS
Age (years)^a^	39 (31–49)	42 (34.2–51.5)	0.0259	41 (34–49.5)	42 (35–51)	NS	36 (28–49)	42 (34–52)	0.0334
Age at onset (years)^a^	26 (21–34.8)	30.2 (24–40)	0.0003	27 (21–34)	29 (24–37)	NS	25 (20–36.8)	33 (26–43)	0.0001
SPMS (%)	26/170 (15.3)	47/257 (18.3)	NS	19/92 (20.7)	34/153 (22.2)	NS	7/78 (9.0)	13/104 (12.5)	NS
Disease duration (years)^a^	9 (5.3–16)	9 (4–14)	NS	10 (6–17)	11 (5–17)	NS	7.5 (4–14.8)	5.5 (3–10.3)	0.0133
EDSS^a^	2.0 (1.0–3.5)	2.0 (1.0–3.5)	NS	2.0 (1.0–3.5)	2.0 (1.0–3.5)	NS	2.0 (1.0–3.0)	2.5 (1.5–4.0)	0.0263
MSSS^a^	2.10 (0.78–5.24)	3.17 (1.16–5.87)	0.0415	1.97 (0.56–5.63)	2.33 (0.88–4.94)	NS	2.28 (1.04–4.97)	4.32 (1.77–6.33)	0.0021
ARR^a^	0.40 (0.22–0.71)	0.43 (0.22–0.85)	NS	0.40 (0.25–0.71)	0.42 (0.22–0.75)	NS	0.37 (0.17–0.71)	0.48 (0.21–0.95)	NS
Barkhof criteria (%)	113/170 (66.5)	205/258 (79.5)	0.0026	69/92 (75.0)	129/155 (83.2)	NS	44/78 (56.4)	76/103 (73.8)	0.0143
Positive OB and/or increased IgG index (%)	58/129 (45.0)	135/200 (67.5)	<0.0001	41/69 (59.4)	89/108 (82.4)	0.0007	17/60 (28.3)	46/92 (50.0)	0.008

The Mann–Whitney *U* test was used to compare continuous variables, and the chi-square test was used to compare categorical variables

*ARR* annualized relapse rate, *EDSS* Kurtzke’s Expanded Disability Status Scale, *MSSS* Multiple Sclerosis Severity Score, *NS* not significant, *OB* oligoclonal IgG bands, *SPMS* secondary progressive multiple sclerosis

^a^Median (interquartile range)

### Comparison of clinical characteristics between HLA-DRB1*15:01-positive and HLA-DRB1*15:01-negative patients

HLA-DRB1*15:01 carriers had a significantly higher frequency of CSF IgG abnormalities than non-DRB1*15:01 carriers (*p* = 0.0058, Additional file 2: Table S9). The effect of DRB1*15:01 on CSF IgG abnormalities was significant in southern patients, but not in northern patients (*p* = 0.0002 and *p* = 0.9042, respectively).

### Comparison of clinical characteristics of HLA-DRB1*04:05- or HLA-DRB1*15:01-positive patients between northern and southern Japanese populations

We compared the clinical features of MS patients with the HLA-DRB1*04:05 or HLA-DRB1*15:01 allele between northern and southern Japan, respectively (Table 4 and Additional file 2: Table S10). MS patients with DRB1*04:05 in northern Japan showed higher frequencies of Barkhof brain lesions and positive CSF IgG abnormalities compared with those in southern Japan (*p* = 0.0105 and *p* = 0.0004). This suggests that higher latitude increases the frequencies of Barkhof brain lesions and CSF IgG abnormalities independent of the HLA-DRB1*04:05 allele. There were no significant differences between northern and southern Japan in the clinical features of MS patients with the DRB1*15:01 allele.

**Table 4:** Clinical characteristics of patients with MS and the HLA-DRB1*04:05 allele

	Northern patients (*n* = 92)	Southern patients (*n* = 80)	*p* value
Number of males/females (ratio)	27/65 (1:2.4)	25/55 (1:2.2)	NS
Age (years)^a^	41 (34–49.5)	36 (28–49)	NS
Age at onset (years)^a^	27 (21–34)	25 (20–36.8)	NS
SPMS (%)	19/92 (20.7)	7/78 (9.0)	0.035
Disease duration (years)^a^	10 (6–17)	7.5 (4–14.8)	NS
EDSS^a^	2.0 (1.0–3.5)	2.0 (1.0–3.0)	NS
MSSS^a^	1.97 (0.56–5.63)	2.28 (1.04–4.97)	NS
ARR^a^	0.40 (0.25–0.71)	0.37 (0.17–0.71)	NS
Barkhof criteria (%)	69/92 (75.0 %)	44/78 (56.4 %)	0.0105
Positive OB and/or increased IgG index (%)	41/69 (59.4 %)	17/60 (28.3 %)	0.0004

The Mann–Whitney *U* test was used to compare continuous variables, and the chi-square test was used to compare categorical variables

*ARR* annualized relapse rate, *EDSS* Kurtzke’s Expanded Disability Status Scale, *MSSS* Multiple Sclerosis Severity Score, *OB* oligoclonal IgG bands, *SPMS* secondary progressive multiple sclerosis

^a^Median (interquartile range)

### Multivariate regression analyses of factors contributing to clinical features and laboratory findings

**Fig. 1:**
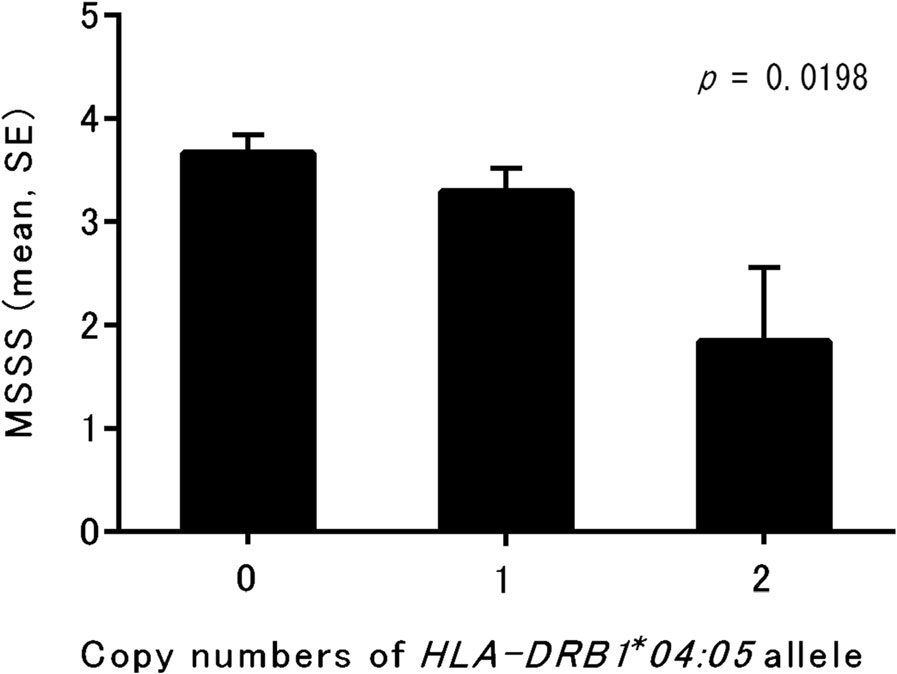
Copy numbers of HLA-DRB1*04:05 allele and MSSS. MSSS is estimated to significantly decrease by 0.57 per DRB1*04:05 allele (*p* = 0.0198), with a possible linear dose effect

We adjusted for factors contributing to age at onset, namely geographic region (northern vs. southern), sex, HLA-DRB1*04:05, and HLA-DRB1*15:01. Only the DRB1*04:05 allele was significantly associated with an earlier age at disease onset (*p* < 0.0001) (Table 5). After adjusting for factors contributing to MSSS, namely geographic region (northern vs. southern), sex, age at onset, MS subtype, ARR, HLA-DRB1*04:05, and HLA-DRB1*15:01, patients living at a higher latitude and with the HLA-DRB1*04:05 allele were independently associated with the lowest quartile of MSSS (adjusted OR = 3.64, *p* < 0.0001 and adjusted OR = 1.81, *p* = 0.0266, respectively). Older age at onset, SPMS, and higher ARR were significantly associated with higher MSSS (*p* = 0.0159, *p* < 0.0001, and *p* < 0.0001, respectively). When the logistic regression model for benign MS was used to adjust for the same confounding factors as MSSS, living at a higher latitude and carrying the HLA-DRB1*04:05 allele were significantly associated with benign MS (adjusted OR = 2.63, *p* = 0.0206; adjusted OR = 2.49, *p* = 0.0275, respectively), while SPMS had a negative association (*p* < 0.0001). Results from the multivariate regression analyses for MSSS and benign MS were consistent with these results. Interestingly, MSSS was estimated to decrease by 0.57 per DRB1*04:05 allele (*p* = 0.0198, Fig. 1), suggesting a gene-dosage effect. A younger age at onset also had a significant positive linear trend with dosage of DRB1*04:05 allele (*p* = 0.0027). After adjusting for the geographic region (northern vs. southern), sex, age at onset, MS subtype, disease duration, ARR, EDSS, HLA-DRB1*04:05, and HLA-DRB1*15:01, we observed a positive correlation between living at a higher latitude and the presence of Barkhof brain lesions (adjusted OR = 1.63, *p* = 0.0434) and a negative correlation between HL-DRB1*04:05 and Barkhof brain lesions (adjusted OR = 0.49, *p* = 0.0043). Additionally, a younger age at onset and SPMS were positively associated with Barkhof brain lesions (*p* = 0.0132 and *p* = 0.0384, respectively). Living at a higher latitude also had a strong positive association with positive OB and/or increased IgG index (adjusted OR = 3.80, *p* < 0.0001). DRB1*04:05 had a strong negative association with CSF IgG abnormalities (and adjusted OR = 0.39, *p* = 0.0004, respectively) after adjusting for geographic region (northern to southern), sex, age at onset, MS subtype, ARR, EDSS, HLA-DRB1*04:05, and HLA-DRB1*15:01.

**Table 5:** Multivariate analysis of factors contributing to clinical and laboratory features

**Age at disease onset**	***β***	***t*** **value**	***p*** **value**
Region (northern Japan)	−0.0918	−1.94	NS
Sex (female)	−0.0127	−0.27	NS
HLA-DRB1*04:05 allele (+)	−0.1970	−4.12	<0.0001
HLA-DRB1*15:01 allele (+)	−0.0239	−0.50	NS
**MSSS (Q1/Q2–Q4)**	**Adjusted OR**	**95 % CI**	***p*** **value**
Region (northern Japan)	3.64	2.15–6.33	<0.0001
Sex (female)	0.98	0.55–1.76	NS
Age at onset	0.97	0.95–0.99	0.0159
SPMS	0.06	0.009–0.20	<0.0001
ARR	0.15	0.07 − 0.31	<0.0001
HLA-DRB1*04:05 allele (+)	1.81	1.07–3.07	0.0266
HLA-DRB1*15:01 allele (+)	1.46	0.83–2.56	NS
**Benign MS**	**Adjusted OR**	**95 % CI**	***p*** **value**
Region (northern Japan)	2.63	1.18–6.09	0.0206
Sex (female)	0.87	0.37–2.00	NS
Age at onset	0.97	0.94–1.01	NS
SPMS	0.04	0.008–0.12	<0.0001
ARR	0.43	0.11–1.42	NS
HLA-DRB1*04:05 allele (+)	2.49	1.12–5.74	0.0275
HLA-DRB1*15:01 allele (+)	1.72	0.76–4.04	NS
**Barkhof criteria**	**Adjusted OR**	**95 % CI**	***p*** **value**
Region (northern Japan)	1.63	1.01–2.63	0.0434
Sex (female)	0.80	0.46–1.35	NS
Age at onset	0.97	0.95–0.99	0.0132
SPMS	2.55	1.05–6.80	0.0384
Disease duration (years)	0.98	0.95–1.01	NS
ARR	1.05	0.67–1.74	NS
EDSS	0.98	0.85–1.13	NS
HLA-DRB1*04:05 allele (+)	0.49	0.30–0.80	0.0043
HLA-DRB1*15:01 allele (+)	1.45	0.85–2.53	NS
**Positive OB and/or increased IgG index**	**Adjusted OR**	**95 % CI**	***p*** **value**
Region (northern Japan)	3.80	2.31–6.36	<0.0001
Sex (female)	1.36	0.78–2.36	NS
Age at onset	1.00	0.98–1.02	NS
SPMS	1.43	0.60–3.51	NS
ARR	0.99	0.66–1.49	NS
EDSS	1.00	0.87–1.16	NS
HLA-DRB1*04:05 allele (+)	0.39	0.23–0.65	0.0004
HLA-DRB1*15:01 allele (+)	1.65	0.95–2.88	NS

Benign MS was defined as an EDSS score **≤** 2.0 at least 10 years after disease onset

*ARR* annualized relapse rate, *CI* confidence interval, *EDSS* Kurtzke’s Expanded Disability Status Scale, *MSSS* Multiple Sclerosis Severity Score, *NS* not significant, *OB* oligoclonal IgG bands, *OR* odds ratio, *Q* quartile, *SPMS* secondary progressive multiple sclerosis

## Discussion

Using multivariate regression analyses, we found that living at a higher latitude and carrying the DRB1*04:05 allele independently lessened the severity of symptoms as evaluated by MSSS. The analyses also revealed that latitude significantly increases the emergence of two hallmarks of MS: fulfillment of the Barkhof criteria for MS, and CSF IgG abnormalities, and that DRB1*04:05 decreases the occurrence of these parameters*.* The difference in clinical features between northern and southern patients was not simply attributable to the difference in the proportion of SPMS patients enrolled, as even after excluding these patients, the difference remained statistically significant.

Thus, DRB1*04:05 appears to decrease MSSS, the frequency of brain lesions meeting the Barkhof criteria, and CSF IgG abnormalities. This is in accordance with our previous report describing DRB1*04:05-positive MS to be a mild disease with lower frequencies of Barkhof brain lesions and CSF abnormalities [6]. DRB1*04 is also associated with OB-negative MS in a Swedish population [27]. Worldwide, the DRB1*04:05 allele is rare in the general population, but it is one of the most common alleles in some select and isolated populations, such as the Japanese, Sardinians, and Papua New Guineans [28]. DRB1*04:05 is a common susceptibility allele for MS in the Japanese population, and DR4 (DRB1*04:05-DQA1*05:01-DQB1*03:01) is also a susceptibility haplotype for MS in the Sardinian population [29], both of which recently showed a steep rise in MS prevalence [10, 30]. We reported that the proportion of DRB1*04:05 carriers in MS patients rapidly increases with descending year of birth [6]. Thus, DRB1*04:05 is a potential risk factor for MS in the younger generation, which is growing up in a modernized environment. Surprisingly, in our study, DRB1*04:05 had a gene-dosage effect on MSSS, decreasing by 0.57 per DRB1*04:05 allele. Therefore, DRB1*04:05 appears to be unique in its ability to both increase susceptibility to MS and decrease its severity as defined by MSSS. It would be of interest to test whether the bidirectional effects of DRB1*04:05 are observed in other populations, such as Sardinians, in which DRB1*04:05 is a common susceptibility allele.

A higher latitude is known to increase MS risk, and this is also true for the Japanese population [25]. It is notable that among Japanese, latitude also has a strong influence on clinical and laboratory manifestations, being positively associated with fulfillment of the Barkhof criteria for MS and CSF IgG abnormalities, but with less severity MS in patients living in northern Japan. In this study, we also found no significant difference in *DRB1* phenotypic frequency between northern and southern controls, suggesting that the observed differences were not likely due to the underlying genetic variation between northern and southern populations. It is interesting to note that latitude also exerts an influence on clinical manifestations in the Japanese population on the mainland with relatively homogeneous *HLA* backgrounds [31]. In New Zealand, a weak inverse relationship between latitude and disease severity has also been observed [32]. Thus, environmental factors related to latitude are assumed to be more important for producing differences in clinical features than *HLA* backgrounds.

The effects of latitude are bidirectional; it decreases disease severity as defined by MSSS while increasing the frequencies of Barkhof brain lesions and CSF IgG abnormalities. These bidirectional effects, in part, mask the effects of DRB1*04:05 on MSSS and fulfillment of Barkhof criteria for MS in northern MS patients. SPMS was associated with greater MSSS, but the frequency of SPMS was smaller in the southern MS population than that in the north. This suggests that the greater severity of MS in the south is not related to a chronic progressive phase. After excluding SPMS patients, greater MSSS scores in the southern group than in the northern became more evident in this study. Therefore, the greater MSSS scores in southern MS patients might be related to neuroinflammation during relapse, as well as poor recovery. Total brain T2-weighted lesion load on MRI has little to no correlation with MS disability [33, 34]. However, gray matter atrophy and cortical lesion burdens are the most significant MRI variables correlating with MS disability [35, 36]. In the future, the measurement of brain parenchymal volume and atrophy, as well as the assessment of cortical demyelination using double-inversion recovery MRI images, are required in our cohort to elucidate the factors influencing the differences in MS severity between the two areas. Although we carefully excluded patients with longitudinally extensive spinal cord lesions, spinal cord lesion load and spinal cord atrophy should also be measured in the future. It is important to clarify the effects of latitude on these parameters.

The clinical features of MS in Asians have been reported to be different to those in patients in Western countries. In the fourth nationwide survey in Japan, SPMS was observed in 15.2 % of patients with conventional (classical)-type MS [10], suggesting that SPMS is less common in Japanese patients than in Western patients. It has repeatedly been reported that Japanese patients with MS have low rates of CSF IgG abnormality and Barkhof brain lesions [10, 21, 37]. Some studies have reported that the presence of OB is associated with a worse MS prognosis in Caucasians [38, 39], while others found no significant difference in prognosis between OB-positive and OB-negative MS patients, including a Japanese cohort [27, 37, 40]. We found that neither the presence of Barkhof brain lesions nor CSF abnormalities was associated with MSSS, suggesting that these factors may not be decisive prognostic factors for MS in Japanese populations. We suggest that living at higher latitude might independently exert stronger effects on MS severity than CSF IgG abnormalities. Indeed, this hypothesis is consistent with findings that higher latitude is associated with milder disability in MS patients in New Zealand [32]. Further research is needed to elucidate other factors related to the association of latitude with MS severity in Japanese populations.

In our study, the difference in disease severity in northern patients, as shown by differences in MSSS between DRB1*04:05 carriers and non-carriers, is not as prominent as in southern patients. In southern patients, lower latitude and an absence of DRB1*04:05 increased the median MSSS to 4.33 in the DRB1*04:05 non-carriers. Conversely, in northern patients, higher latitude and the presence of DRB1*04:05 decreased MSSS to 1.97 in DRB1*04:05 carriers. Because the latter value is close to the lower limit, this lowering effect may not be obvious. Alternatively, the effects of latitude might be more potent in DRB1*04:05 non-carriers than in DRB1*04:05 carriers. Further studies are needed to investigate the interactions between the two factors in larger cohorts.

The DRB1*15:01 allele is a strong genetic risk factor for MS in Caucasians and has been associated with earlier age at onset [4] and OB positivity [41]. In the present study, DRB1*15:01 was associated with CSF IgG abnormalities and marginally correlated with fulfillment of the Barkhof criteria for MS. However, these associations lost significance after adjusting for contributing factors. Thus, the effects of DRB1*15:01 on clinical manifestations may be smaller in Asian populations than in European populations.

We also found that DRB1*01:01, DRB1*09:01, DRB1*13:02, and DRB1*15:02 were protective alleles in the Japanese MS population. Based on a meta-analysis, DRB1*09:01 has been reported to be a protective allele for MS in a Chinese population, and this allele is more common in Asians than in other ethnic groups [42]. DRB1*01:01, DRB1*13:02, and DRB1*15:02 have been determined to be novel protective alleles for MS in Japanese populations by the present study. Among the Basque, a Caucasian population living in northern Spain and southwestern France, the DRB1*01:01 allele has a negative association with the disease [43]. The DRB1*15:02-DQB1*06:01 haplotype also decreases the risk for MS in a Sardinian population [44]. Interestingly, DRB1*15:01 and DRB1*15:02 only differ at amino acid position 86 (a valine for DRB1*15:01 and a glycine for DRB1*15:02). The difference of only one amino acid results in opposite effects on MS susceptibility. DRB1*15:01, which contains valine, only tolerates small residues, while DRB1*15:02, which contains glycine, can accommodate large aromatic peptide residues [45]. This small difference may determine the characteristic of presenting antigens, thereby resulting in opposite effects on MS.

Our study has some limitations; first, we did not measure the effects of disease-modifying drugs (DMDs) on clinical course. However, it would be difficult to explain the differences in the clinical manifestations and disease course according to regional and genetic factors based on DMD usage alone as such factors could not be taken into account in the use of DMDs in daily clinical practice. Second, we could not study the interactions between *HLA-DRB1* and environmental risk factors because of the small study sample size. The interaction between DRB1*15:01 and environmental risk factors, such as smoking, obesity, vitamin D, and Epstein–Barr virus infection, is well known [46–48]; any interaction between DRB1*04:05 and these environmental risk factors remains to be established. Third, although this is the largest study comparing clinical manifestations and genetic backgrounds between patients residing in northern and southern Japan, the sample size was still smaller than in Caucasian MS studies. Nevertheless, we believe it would be valuable to make further regional comparisons of the clinical manifestations and disease courses in genetically and culturally homogeneous populations residing in a wide range of latitudes to shed more light on the possible genetic and environmental influences on neuroinflammation.

## Conclusions

Living at a higher latitude and carrying the DRB1*04:05 allele reduces the severity of MS in Japanese MS populations as defined by MSSS. However, both factors influence the frequencies of Barkhof brain lesions and CSF IgG abnormalities in different ways; that is, higher latitudes increase the frequencies of Barkhof brain lesions and CSF IgG abnormalities, whereas DRB1*04:05 decreases them. Further large-scale studies focusing on genetic-environmental interactions are needed to determine the factors associated with the severity of neuroinflammation in MS.

## Additional files

Additional file 1: Figure S1. Map of Japanese main islands. The main islands of the Japanese archipelago span 31–46° north [10]. The residing areas of northern MS patients recruited from the Hokkaido Island are located 42–45° north, while those of southern MS patients recruited from the southern half of the Japanese archipelago span 33–35° north. Thus, there are at least 7° of difference between the residing areas of the northern and southern patients in the present study. (TIF 121 kb) Additional file 2: 
**Table S1.** Phenotypic frequencies of *HLA-DPB1* alleles in patients with MS. **Table S2.** Phenotypic frequencies of *HLA-DRB1* alleles in MS patients from northern Japan. **Table S3.** Phenotypic frequencies of *HLA-DRB1* alleles in MS patients from southern Japan. **Table S4.** Phenotypic frequencies of *HLA-DRB1* alleles in HCs. **Table S5.** Demographic features of patients with MS, excluding SPMS, according to region. **Table S6.** Comparison of MS demographic features between northern and southern patients using clinical data from southern patients followed up until January 2013. **Table S7.** Comparison of MS demographic features between northern and southern patients using clinical data from southern patients followed up until January 2013, excluding SPMS. **Table S8.** Comparison of MSSS between MS patients with and without Barkhof brain lesions, and between those with and without CSF IgG abnormalities. **Table S9.** Comparison of clinical features in patients with MS according to the presence or absence of *HLA-DRB1*15:01.* **Table S10.** Clinical characteristics of MS patients with the *HLA-DRB1*15:01* allele. (DOCX 54 kb)

## Abbreviations

CI: Confidence interval
CSF: Cerebrospinal fluid
DMDs: Disease-modifying drugs
EDSS: Expanded Disability Status Scale
HCs: Healthy controls
*HLA*: Human leukocyte antigen
MS: Multiple sclerosis
MSSS: Multiple Sclerosis Severity Score
NA: Not applicable
NS: Not significant
OB: Oligoclonal IgG bands
OR: Odds ratio
SPMS: Secondary progressive multiple sclerosis
