# Supplementary figures and images for: Latitude and HLA-DRB1*04:05 independently influence disease severity in Japanese multiple sclerosis: a cross-sectional study
Source: J Neuroinflammation. 2016 Sep 6;13(1):239. doi: 10.1186/s12974-016-0695-3 (PMC5013608; doi:10.1186/s12974-016-0695-3)

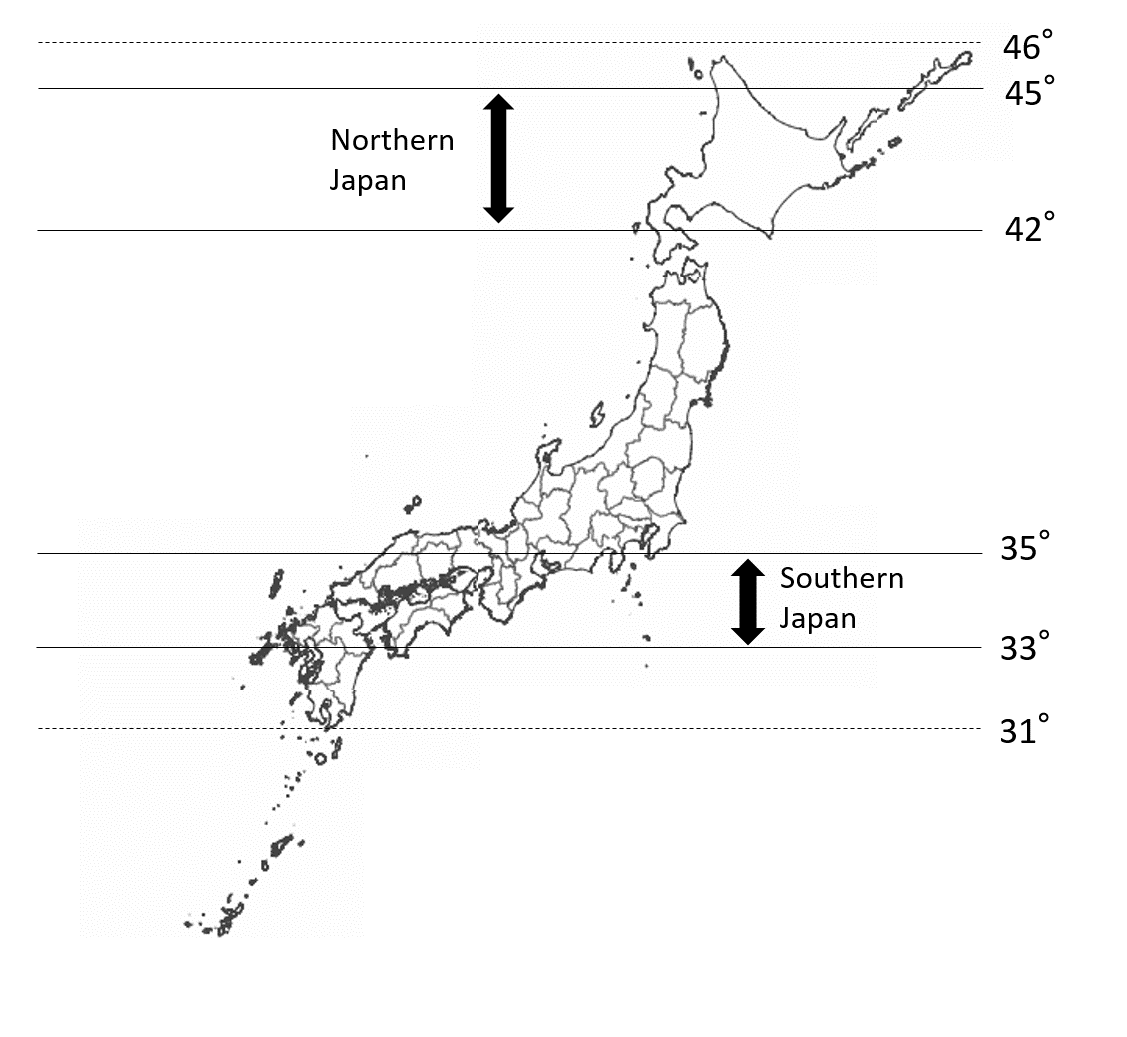

Supplement: Additional file 1: Figure S1. — Map of Japanese main islands. The main islands of the Japanese archipelago span 31–46° north [10]. The residing areas of northern MS patients recruited from the Hokkaido Island are located 42–45° north, while those of southern MS patients recruited from the southern half of the Japanese archipelago span 33–35° north. Thus, there are at least 7° of difference between the residing areas of the northern and southern patients in the present study. (TIF 121 kb) [file 12974_2016_695_MOESM1_ESM.tif]
