# Supplementary material for: Latitude and HLA-DRB1*04:05 independently influence disease severity in Japanese multiple sclerosis: a cross-sectional study
Source: J Neuroinflammation. 2016 Sep 6;13(1):239. doi: 10.1186/s12974-016-0695-3 (PMC5013608; doi:10.1186/s12974-016-0695-3)
Supplement: Additional file 2: — Table S1. Phenotypic frequencies of HLA-DPB1 alleles in patients with MS. Table S2. Phenotypic frequencies of HLA-DRB1 alleles in MS patients from northern Japan. Table S3. Phenotypic frequencies of HLA-DRB1 alleles in MS patients from southern Japan. Table S4. Phenotypic frequencies of HLA-DRB1 alleles in HCs. Table S5. Demographic features of patients with MS, excluding SPMS, according to region. Table S6. Comparison of MS demographic features between northern and southern patients using clinical data from southern patients followed up until January 2013. Table S7. Comparison of MS demographic features between northern and southern patients using clinical data from southern patients followed up until January 2013, excluding SPMS. Table S8. Comparison of MSSS between MS patients with and without Barkhof brain lesions, and between those with and without CSF IgG abnormalities. Table S9. Comparison of clinical features in patients with MS according to the presence or absence of HLA-DRB1*15:01. Table S10. Clinical characteristics of MS patients with the HLA-DRB1*15:01 allele. (DOCX 54 kb) [file 12974_2016_695_MOESM2_ESM.docx]

**Table S1.** Phenotypic frequencies of *HLA-DPB1* alleles in patients with MS

| **Allele** | **Phenotype frequency, n (%)** | | **OR** | **95% CI** | ***p^uncorr^*** | ***p^corr^*** |
| --- | --- | --- | --- | --- | --- | --- |
|  | **MS**  (n = 434) | **HCs**  (n = 394) |  |  |  |  |
| *02:01* | 191 (44.0) | 148 (37.6) | 1.31 | 0.99–1.73 | 0.0596 | NS |
| *02:02* | 27 (6.2) | 21 (5.3) | 1.18 | 0.65–2.12 | 0.5836 | NS |
| *03:01* | 46 (10.6) | 25 (6.4) | 1.75 | 1.05–2.91 | 0.0290 | NS |
| *04:01* | 18 (4.2) | 36 (9.1) | 0.43 | 0.24–0.77 | 0.0037 | NS |
| *04:02* | 67 (15.4) | 73 (18.5) | 0.80 | 0.56–1.16 | 0.2361 | NS |
| *05:01* | 298 (68.7) | 254 (64.5) | 1.21 | 0.90–1.61 | 0.2008 | NS |
| *06:01* | 5 (1.2) | 8 (2.0) | 0.56 | 0.18–1.73 | 0.4043 | NS |
| *09:01* | 61 (14.1) | 70 (17.8) | 0.76 | 0.52–1.10 | 0.1439 | NS |
| *13:01* | 17 (3.9) | 17 (4.3) | 0.90 | 0.46–1.80 | 0.7734 | NS |
| *14:01* | 11 (2.5) | 11 (2.8) | 0.91 | 0.39–2.11 | 0.8181 | NS |
| *17:01* | 2 (0.5) | 0 | NA | NA | 0.5006 | NS |
| *19:01* | 2 (0.5) | 4 (1.0) | 0.45 | 0.08–2.48 | 0.4317 | NS |
| *22:01* | 0 | 1 (0.3) | 0 | NA | 0.4758 | NS |
| *41:01* | 0 | 2 (0.5) | 0 | NA | 0.2261 | NS |

CI = confidence interval, HCs = healthy controls, MS = multiple sclerosis, NA = not applicable, NS = not significant, OR = odds ratio.

*p^corr^* = corrected *p*-value.

*p^uncorr^* was corrected by multiplying the value by 14 to calculate *p^corr^*.

**Table S2.** Phenotypic frequencies of *HLA-DRB1* alleles in MS patients from northern Japan

| **Allele** | **Phenotype frequency**, n (%) | | **OR** | **95% CI** | ***p^uncorr^*** | ***p^corr^*** |
| --- | --- | --- | --- | --- | --- | --- |
|  | **MS**  (n = 247) | **HCs**  (n = 159) |  |  |  |  |
| *01:01* | 8 (3.2) | 18 (11.3) | 0.26 | 0.11–0.62 | 0.0012 | **0.0384** |
| *03:03* | 1 (0.4) | 0 | NA | NA | 1.0000 | NS |
| *04:01* | 4 (1.6) | 6 (3.8) | 0.42 | 0.12–1.51 | 0.1992 | NS |
| *04:03* | 31 (12.6) | 10 (6.3) | 2.14 | 1.02–4.49 | 0.0410 | NS |
| *04:04* | 2 (0.8) | 2 (1.3) | 0.64 | 0.09–4.60 | 0.6463 | NS |
| *04:05* | 92 (37.3) | 34 (21.4) | 2.18 | 1.38–3.45 | 0.0007 | **0.0224** |
| *04:06* | 19 (7.7) | 13 (8.2) | 0.94 | 0.45–1.95 | 0.8598 | NS |
| *04:07* | 2 (0.8) | 2 (1.3) | 0.64 | 0.09–4.60 | 0.6463 | NS |
| *04:10* | 13 (5.3) | 5 (3.1) | 1.71 | 0.60–4.90 | 0.4594 | NS |
| *07:01* | 1 (0.4) | 2 (1.3) | 0.32 | 0.03–3.55 | 0.5637 | NS |
| *08:01* | 1 (0.4) | 1 (0.6) | 0.64 | 0.04–10.34 | 1.0000 | NS |
| *08:02* | 23 (9.3) | 15 (9.4) | 0.99 | 0.50–1.95 | 0.9671 | NS |
| *08:03* | 35 (14.2) | 23 (14.5) | 0.98 | 0.55–1.72 | 0.9338 | NS |
| *09:01* | 49 (19.8) | 42 (26.4) | 0.69 | 0.43–1.10 | 0.1209 | NS |
| *10:01* | 2 (0.8) | 0 | NA | NA | 0.5223 | NS |
| *11:01* | 11 (4.5) | 9 (5.7) | 0.78 | 0.31–1.92 | 0.6414 | NS |
| *12:01* | 13 (5.3) | 14 (8.8) | 0.58 | 0.26–1.26 | 0.1621 | NS |
| *12:02* | 3 (1.2) | 5 (3.1) | 0.38 | 0.09–1.61 | 0.2717 | NS |
| *13:01* | 2 (0.8) | 1 (0.6) | 1.29 | 0.12–14.34 | 1.0000 | NS |
| *13:02* | 11 (4.5) | 23 (14.5) | 0.28 | 0.13–0.58 | 0.0004 | **0.0128** |
| *13:07* | 0 | 1 (0.6) | 0 | NA | 0.3916 | NS |
| *14:01* | 3 (1.2) | 0 | NA | NA | 0.2835 | NS |
| *14:02* | 1 (0.4) | 1 (0.6) | 0.64 | 0.04–10.34 | 1.0000 | NS |
| *14:03* | 8 (3.2) | 8 (5.0) | 0.63 | 0.23–1.72 | 0.4358 | NS |
| *14:05* | 10 (4.1) | 4 (2.5) | 1.64 | 0.50–5.31 | 0.5794 | NS |
| *14:06* | 7 (2.8) | 2 (1.3) | 2.29 | 0.47–11.16 | 0.4922 | NS |
| *14:07* | 0 | 2 (1.3) | 0 | NA | 0.1528 | NS |
| *14:54* | 14 (5.7) | 10 (6.3) | 0.90 | 0.39–2.07 | 0.8312 | NS |
| *15:01* | 75 (30.4) | 27 (17.0) | 2.13 | 1.30–3.50 | 0.0024 | NS |
| *15:02* | 33 (13.4) | 28 (17.6) | 0.72 | 0.42–1.25 | 0.2421 | NS |
| *15:10* | 1 (0.4) | 0 | NA | NA | 1.0000 | NS |
| *16:02* | 3 (1.2) | 2 (1.3) | 0.97 | 0.16–5.84 | 1.0000 | NS |

CI = confidence interval, HCs = healthy controls, MS = multiple sclerosis, NA = not applicable, NS = not significant, OR = odds ratio.

*p^corr^* = corrected *p*-value.

*p^uncorr^* was corrected by multiplying the value by 32 to calculate *p^corr^*.

**Table S3.** Phenotypic frequencies of *HLA-DRB1* alleles in MS patients from southern Japan

| **Allele** | **Phenotype frequency**, n (%) | | **OR** | **95% CI** | ***p^uncorr^*** | ***p^corr^*** |
| --- | --- | --- | --- | --- | --- | --- |
|  | **MS**  (n = 187) | **HCs**  (n = 235) |  |  |  |  |
| *01:01* | 16 (8.6) | 35 (14.9) | 0.53 | 0.29–1.00 | 0.0472 | NS |
| *04:01* | 3 (1.6) | 3 (1.3) | 1.26 | 0.25–6.32 | 1.0000 | NS |
| *04:03* | 11 (5.9) | 12 (5.1) | 1.16 | 0.50–2.69 | 0.7272 | NS |
| *04:05* | 80 (42.8) | 66 (28.1) | 1.91 | 1.28–2.87 | 0.0016 | **0.0384** |
| *04:06* | 23 (12.3) | 13 (5.5) | 2.39 | 1.18–4.87 | 0.0134 | NS |
| *04:07* | 0 | 2 (0.9) | 0 | NA | 0.5053 | NS |
| *04:10* | 11 (5.9) | 3 (1.3) | 4.83 | 1.33–17.59 | 0.0119 | NS |
| *07:10* | 1 (0.5) | 0 | NA | NA | 0.4431 | NS |
| *08:02* | 15 (8.0) | 14 (6.0) | 1.38 | 0.65–2.93 | 0.4051 | NS |
| *08:03* | 28 (15.0) | 33 (14.0) | 1.08 | 0.63–1.86 | 0.7871 | NS |
| *09:01* | 23 (12.3) | 62 (26.4) | 0.39 | 0.23–0.66 | 0.0003 | **0.0072** |
| *10:01* | 0 | 2 (0.9) | 0 | NA | 0.5053 | NS |
| *11:01* | 7 (3.7) | 10 (4.3) | 0.88 | 0.33–2.34 | 1.0000 | NS |
| *12:01* | 13 (7.0) | 24 (10.2) | 0.66 | 0.32–1.33 | 0.2394 | NS |
| *12:02* | 2 (1.1) | 10 (4.3) | 0.24 | 0.05–1.12 | 0.0743 | NS |
| *13:01* | 1 (0.5) | 1 (0.4) | 1.26 | 0.08–20.25 | 1.0000 | NS |
| *13:02* | 9 (4.8) | 23 (9.8) | 0.47 | 0.21–1.03 | 0.0552 | NS |
| *14:03* | 12 (6.4) | 6 (2.6) | 2.62 | 0.96–7.11 | 0.0563 | NS |
| *14:05* | 7 (3.7) | 11 (4.7) | 0.79 | 0.30–2.08 | 0.8093 | NS |
| *14:06* | 4 (2.1) | 4 (1.7) | 1.26 | 0.31–5.12 | 0.7370 | NS |
| *14:54* | 6 (3.2) | 18 (7.7) | 0.40 | 0.16–1.03 | 0.0499 | NS |
| *15:01* | 50 (26.7) | 40 (17.0) | 1.78 | 1.11–2.85 | 0.0155 | NS |
| *15:02* | 22 (11.8) | 55 (23.4) | 0.44 | 0.25–0.75 | 0.0021 | NS |
| *16:02* | 1 (0.5) | 2 (0.9) | 0.63 | 0.06–6.96 | 1.0000 | NS |

CI = confidence interval, HCs = healthy controls, MS = multiple sclerosis, NA = not applicable, NS = not significant, OR = odds ratio.

*p^corr^* = corrected *p*-value.

*p^uncorr^* was corrected by multiplying the value by 24 to calculate *p^corr^*.

**Table S4.** Phenotypic frequencies of *HLA-DRB1* alleles in HCs

| **Allele** | **Phenotype frequency**, n (%) | | ***p^uncorr^*** | ***p^corr^*** |
| --- | --- | --- | --- | --- |
|  | **Northern HCs**  (n = 159) | **Southern HCs**  (n = 235) |  |  |
| *01:01* | 18 (11.3) | 35 (14.9) | 0.3079 | NS |
| *04:01* | 6 (3.8) | 3 (1.3) | 0.1663 | NS |
| *04:03* | 10 (6.3) | 12 (5.1) | 0.6583 | NS |
| *04:04* | 2 (1.3) | 0 | 0.1622 | NS |
| *04:05* | 34 (21.4) | 66 (28.1) | 0.1337 | NS |
| *04:06* | 13 (8.2) | 13 (5.5) | 0.2996 | NS |
| *04:07* | 2 (1.3) | 2 (0.9) | 1.0000 | NS |
| *04:10* | 5 (3.1) | 3 (1.3) | 0.2772 | NS |
| *07:01* | 2 (1.3) | 0 | 0.1622 | NS |
| *08:01* | 1 (0.6) | 0 | 0.4036 | NS |
| *08:02* | 15 (9.4) | 14 (6.0) | 0.1948 | NS |
| *08:03* | 23 (14.5) | 33 (14.0) | 0.9061 | NS |
| *09:01* | 42 (26.4) | 62 (26.4) | 0.9943 | NS |
| *10:01* | 0 | 2 (0.9) | 0.5174 | NS |
| *11:01* | 9 (5.7) | 10 (4.3) | 0.6330 | NS |
| *12:01* | 14 (8.8) | 24 (10.2) | 0.6424 | NS |
| *12:02* | 5 (3.1) | 10 (4.3) | 0.7895 | NS |
| *13:01* | 1 (0.6) | 1 (0.4) | 1.0000 | NS |
| *13:02* | 23 (14.5) | 23 (9.8) | 0.1560 | NS |
| *13:07* | 1 (0.6) | 0 | 0.4036 | NS |
| *14:02* | 1 (0.6) | 0 | 0.4036 | NS |
| *14:03* | 8 (5.0) | 6 (2.6) | 0.2670 | NS |
| *14:05* | 4 (2.5) | 11 (4.7) | 0.4216 | NS |
| *14:06* | 2 (1.3) | 4 (1.7) | 1.0000 | NS |
| *14:07* | 2 (1.3) | 0 | 0.1622 | NS |
| *14:54* | 10 (6.3) | 18 (7.7) | 0.6035 | NS |
| *15:01* | 27 (17.0) | 40 (17.0) | 0.9917 | NS |
| *15:02* | 28 (17.6) | 55 (23.4) | 0.1664 | NS |
| *16:02* | 2 (1.3) | 2 (0.9) | 1.0000 | NS |

HCs = healthy controls, NS = not significant.

*p^corr^* = corrected *p*-value.

*p^uncorr^* was corrected by multiplying the value by 24 to calculate *p^corr^.*

**Table S5.** Demographic features of patients with MS, excluding SPMS, according to region

|  | **Northern patients**  (n = 194) | **Southern patients**  (n = 167) | ***p*-value** |
| --- | --- | --- | --- |
| Number of males/females (ratio) | 45/149 (1:3.3) | 46/121 (1:2.6) | NS |
| Age (years) ^a^ | 40 (33–47) | 40 (32–50) | NS |
| Age at onset (years) ^a^ | 29.5 (22–36) | 30 (23-40) | NS |
| Disease duration (years) ^a^ | 9 (5–14) | 6 (3–11) | **0.0004** |
| EDSS ^a^ | 1.5 (1–2.5) | 2 (1–3) | **0.0008** |
| MSSS ^a^ | 1.45 (0.53–3.67) | 3.34 (1.28–5.38) | **< 0.0001** |
| ARR ^a^ | 0.43 (0.25–0.75) | 0.43 (0.19–0.91) | NS |
| Barkhof criteria (%) | 148/194 (76.3%) | 105/161 (65.2%) | **0.0217** |
| Positive OB and/or increased IgG index (%) | 103/141 (73.1%) | 53/135 (39.3%) | **< 0.0001** |
| Phenotypic frequency of *HLA-DRB1*04:05* (%) | 73 (37.6) | 73 (43.7) | NS |
| Phenotypic frequency of *HLA-DRB1*15:01* (%) | 60 (30.9) | 46 (27.5) | NS |

ARR = annualized relapse rate, EDSS = Kurtzke’s Expanded Disability Status Scale, MSSS = Multiple Sclerosis Severity Score, OB = oligoclonal IgG bands, SPMS = secondary progressive multiple sclerosis.

^a^ Median (interquartile range).

The Mann–Whitney U-test was used to compare continuous variables, and the chi-square test was used to compare categorical variables.

**Table S6.** Comparison of MS demographic features between northern and southern patients using clinical data from southern patients followed up until January 2013

|  | **Northern patients**  (n = 247) | **Southern patients**  (n = 124) | ***p*-value** |
| --- | --- | --- | --- |
| Number of males/females (ratio) | 62/185 (1:3.0) | 35/89 (1:2.5) | NS |
| Age (years) ^a^ | 41 (34–50) | 42 (32–53) | NS |
| SPMS (ratio) | 53/245 (21.6%) | 15/124 (12.1%) | **0.0256** |
| Disease duration (years) ^a^ | 10 (6–17) | 9 (4–14.8) | NS |
| EDSS ^a^ | 2 (1–3.5) | 2 (1–3) | NS |
| MSSS ^a^ | 2.13 (0.69–5.24) | 2.44 (0.94–4.83) | NS |

EDSS = Kurtzke’s Expanded Disability Status Scale, MSSS = Multiple Sclerosis Severity Score, NS = not significant, SPMS = secondary progressive multiple sclerosis.

^a^ Median (interquartile range).

The Mann–Whitney U-test was used to compare continuous variables, and the chi-square test was used to compare categorical variables.

**Table S7.** Comparison of MS demographic features between northern and southern patients using clinical data from southern patients followed up until January 2013, excluding SPMS

|  | **Northern patients**  (n = 194) | **Southern patients**  (n = 109) | ***p*-value** |
| --- | --- | --- | --- |
| Number of males/females (ratio) | 45/149 (1:3.3) | 25/84 (1:3.4) | NS |
| Age (years) ^a^ | 40 (33–47) | 42 (32–53) | NS |
| Disease duration (years) ^a^ | 9 (5-14) | 8 (4-13.5) | NS |
| EDSS ^a^ | 1.5 (1-2.5) | 1 (1-2.5) | NS |
| MSSS ^a^ | 1.45 (0.53-3.67) | 2.23 (0.72-4.30) | **0.0411** |

EDSS = Kurtzke’s Expanded Disability Status Scale, MSSS = Multiple Sclerosis Severity Score, NS = not significant, SPMS = secondary progressive multiple sclerosis.

^a^ Median (interquartile range).

The Mann–Whitney U-test was used to compare continuous variables, and the chi-square test was used to compare categorical variables.

**Table S8.** Comparison of MSSS between MS patients with and without Barkhof brain lesions, and between those with and without CSF IgG abnormalities

|  | **Positive Barkhof brain lesions** | **Negative Barkhof brain lesions** | ***p*-value** |
| --- | --- | --- | --- |
| Total patients (n = 425) | 2.60 (1.04–5.74)  (n = 315) | 2.73 (0.67–5.38)  (n = 110) | NS |
| Northern patients (n =245 ) | 2.33 (0.78–5.57)  (n = 196) | 1.45 (0.41–4.83)  (n = 49) | NS |
| Southern patients (n = 180) | 3.54 (1.45–5.87)  (n = 119) | 3.69 (0.87–6.40)  (n = 61) | NS |
|  | **Positive OB and/or increased IgG index** | **Negative OB and/or increased IgG index** | ***p*-value** |
| Total patients (n = 327) | 3.45 (1.41–5.80)  (n = 192) | 2.44 (0.88–5.74)  (n = 135) | NS |
| Northern patients (n = 175) | 2.44 (0.63–5.43)  (n = 129) | 1.52 (0.48–4.97)  (n = 46) | NS |
| Southern patients (n = 152) | 4.57 (2.01–6.33)  (n = 63) | 3.46 (1.08–6.08)  (n = 89) | NS |

MSSS = Multiple Sclerosis Severity Score, NS = not significant, OB = oligoclonal IgG bands.

Values of MSSS are presented as median (interquartile range).

The Mann–Whitney U-test was used to compare MSSS.

**Table S9.** Comparison of clinical features in patients with MS according to the presence or absence of *HLA-DRB1*15:01*

| **DRB1** | **Total patients** | | | **Northern patients** | | | | **Southern patients** | | |
| --- | --- | --- | --- | --- | --- | --- | --- | --- | --- | --- |
|  | ***15:01* (+)**  **(n = 125)** | ***15:01* (−)**  **(n = 309)** | ***p-*value** | ***15:01* (+)**  **(n = 75)** | ***15:01* (−)**  **(n = 172)** | ***p*-value** | ***15:01* (+)**  **(n = 50)** | | ***15:01* (−)**  **(n = 137)** | ***p*-value** |
| Number of males/females (ratio) | 27/98 (1:3.6) | 93/216 (1:2.3) | NS | 18/57 (1:3.2) | 44/128 (1:2.9) | NS | 9/41(1:4.6) | | 49/88 (1:1.8) | **0.0201** |
| Age (years) ^a^ | 42 (34.5–48) | 40 (33–51) | NS | 43 (37–49) | 40.5 (33–50.8) | NS | 41 (29–46.3) | | 39 (32–51) | NS |
| Age at onset (years) ^a^ | 30 (23–38) | 29 (22–39) | NS | 30 (25–38) | 28 (21–35) | NS | 29.5 (20–38) | | 30(23–42) | NS |
| SPMS (%) | 19/124 (15.3) | 54/303 (17.8) | NS | 15/74 (20.3) | 38/171 (22.2) | NS | 4/50 (8.0) | | 16/132 (12.1) | NS |
| Disease duration (years) ^a^ | 9 (4–16) | 9 (5–15) | NS | 10 (4–17) | 10.5 (6–17) | NS | 7 (2.8–15) | | 6 (3–12) | NS |
| EDSS ^a^ | 2.0 (1.0–3.25) | 2.0 (1.0–3.5) | NS | 2.0 (1.0–3.5) | 2.0 (1.0–3.5) | NS | 2.0 (1.5–3.0) | | 2.0 (1.0–3.5) | NS |
| MSSS ^a^ | 2.34 (0.78–5.31) | 2.82 (1.02–5.74) | NS | 1.92 (0.58–5.02) | 2.34 (0.80–5.29) | NS | 3.69 (1.45–5.50) | | 3.50 (1.32–6.01) | NS |
| ARR ^a^ | 0.42 (0.23–0.87) | 0.40 (0.21–0.74) | NS | 0.41 (0.22–0.77) | 0.41 (0.25–0.71) | NS | 0.50 (0.27–0.99) | | 0.39 (0.17–0.82) | NS |
| Barkhof criteria (%) | 99/123 (80.5) | 219/305 (71.8) | NS | 62/75 (82.7) | 136/172 (79.1) | NS | 37/48 (77.1) | | 83/133 (62.4) | NS |
| Positive OB and/or increased IgG index (%) | 70/100 (70.0) | 123/229 (53.7) | **0.0058** | 43/59 (72.9) | 87/118 (73.7) | NS | 27/41 (65.9) | | 36/111 (32.4) | **0.0002** |

ARR = annualized relapse rate, EDSS = Kurtzke’s Expanded Disability Status Scale, MSSS = Multiple Sclerosis Severity Score, NS = not significant, OB = oligoclonal IgG bands, SPMS = secondary progressive multiple sclerosis.

^a^ Median (interquartile range)

The Mann–Whitney U-test was used to compare continuous variables, and the chi-square test was used to compare categorical variables.

**Table S10.** Clinical characteristics of MS patients with the *HLA-DRB1*15:01* allele

|  | **Northern patients**  (n = 75) | **Southern patients**  (n = 50) | ***p*-value** |
| --- | --- | --- | --- |
| Number of males/females (ratio) | 18/57 (1:3.2) | 9/41 (1:4.6) | NS |
| Age (years) ^a^ | 43 (37–49) | 41 (29–46.3) | NS |
| Age at onset (years) ^a^ | 30 (25–38) | 29.5 (20–38) | NS |
| SPMS (%) | 15/74 (20.3) | 4/50 (8.0) | NS |
| Disease duration (years) ^a^ | 10 (4–17) | 7 (2.8–15) | NS |
| EDSS ^a^ | 2.0 (1.0–3.5) | 2.0 (1.5–3.0) | NS |
| MSSS ^a^ | 1.92 (0.58–5.02) | 3.69 (1.45–5.50) | NS |
| ARR ^a^ | 0.41 (0.22–0.77) | 0.50 (0.27–0.99) | NS |
| Barkhof criteria (%) | 62/75 (82.7%) | 37/48 (77.1%) | NS |
| Positive OB and/or increased IgG index (%) | 43/59 (72.9%) | 27/41 (65.9%) | NS |

ARR = annualized relapse rate, EDSS = Kurtzke’s Expanded Disability Status Scale, MSSS = Multiple Sclerosis Severity Score, OB = oligoclonal IgG bands, SPMS = secondary progressive multiple sclerosis.

^a^ Median (interquartile range).

The Mann–Whitney U-test was used to compare continuous variables, and the chi-square test was used to compare categorical variables.
